# Effects of different physical exercise programs on psychological stress and group cohesion in children

**DOI:** 10.3389/fpsyg.2026.1837922

**Published:** 2026-08-06

**Authors:** Kaixin Niu, Gang Chen, Guiming Zhu, Miyu Wang, Pengfei Li, Sheng Zhou, Rongbin Yin

**Affiliations:** 1 Physical Education and Sports School, Soochow University, Suzhou, China; 2 Department of Basic Course, Suzhou City University, Suzhou, China

**Keywords:** physical exercise, psychological stress, group cohesion, children, interactive programs, coactive programs

## Abstract

**Purpose:**

This study aimed to examine the effects of different physical exercise programs on psychological stress and group cohesion in children aged 10–11 years and to test the mediating role of group cohesion in the relationship between physical exercise and psychological stress.

**Methods:**

A total of 144 fifth-grade students aged 10–11 years were recruited from four administrative classes at a primary school in Suzhou using cluster sampling. The four classes were randomly allocated to Interactive Programs (*n* = 73) and Coactive Programs (*n* = 71). The participants’ psychological stress and group cohesion levels were measured before and after the 16-week physical exercise intervention. Data analysis employed repeated measures ANOVA, correlation analysis, and mediation effect testing.

**Results:**

(1) Both types of physical exercise significantly reduced psychological stress levels and enhanced group cohesion among children aged 10–11 years (*P* < 0.01). (2) Compared with the coactive program group, the interactive program group showed significantly greater improvements in both psychological stress reduction and group cohesion (*P* < 0.01). (3) Participation in interactive physical activities is a significant negatively predictive factor for psychological stress and a significant positively predictive factor for group cohesion. Group cohesion is significantly negatively correlated with psychological stress. (4) The mediating role of group cohesion in the relationship between interactive exercise programs based on interaction and psychological stress did not reach statistical significance.

**Conclusion:**

Physical exercise can effectively alleviate psychological stress and improve group cohesion in children aged 10–11 years. Notably, interactive programs are more effective than coactive programs in promoting both outcomes. The impact of interactive programs on psychological stress is primarily achieved through a direct pathway, with group cohesion not playing a significant mediating role.

## 1 Introduction

Landers et al. proposed that the degree of cooperative effort and interdependence required in different sports can be explained using an interactive-coactive continuum (Landers and Lüschen, 1974). Physical exercise programs are divided into interactive and coactive categories based on this structure (Ji et al., 2016). Interactive programs are those in which achieving excellence requires close collaboration and teamwork. Collaboration, trust, and group cohesion are essential in these situations. Coactive programs, conversely, involve minimal interaction among participants. Individual abilities, physical fitness, and skills are the main factors determining success, and goals are mostly accomplished by individual effort rather than teamwork.

Physical activity (PA) has been extensively studied and proven effective as a non-pharmacological intervention for children’s mental health. According to a biological study, children’s hypothalamic-pituitary-adrenal axis responses are influenced by physical activity, which enhances mental health (Martikainen et al., 2013). Numerous studies show that PA improves self-esteem, lowers rates of anxiety and depression, and even helps students perform better academically (Biddle and Asare, 2011). But not every activity has the same positive effects on mental health. In a study involving more than 10,000 American kids, Hoffmann et al. discovered that kids who played team sports like basketball and soccer had reduced rates of psychological problems like social withdrawal, anxiety, and depression. Conversely, children engaged in individual sports like tennis and gymnastics showed increased scores on related symptoms (Hoffmann et al., 2022). Individuals who participate in solitary sports are more likely than those who participate in team sports to experience detrimental mental health symptoms, including anxiety and depression, according to numerous studies on child and adult athletes (Pluhar et al., 2019; Reardon and Hitchcock, 2024). This discrepancy emphasizes how sport type influences mental health results.

Physical exercise, as an effective means of alleviating psychological stress, has had its stress-reducing mechanisms validated through multiple studies. Exercise significantly reduces anxiety and tension by inhibiting stress-induced cardiovascular reactions and stimulating the production of endorphins in the neurological system (Salmon, 2001). Academic pressure was positively connected with depression and anxiety, but both were adversely correlated with physical activity, according to a study of 1,533 teenagers in eastern China (Zhu et al., 2021). Significant positive relationships between physical activity, self-efficacy, stress self-management, and mental health were found when Zhang, G., examined survey data from 400 middle school students (Zhang et al., 2024). Adolescents’ mental health is generally greatly improved by physical exercise interventions in a number of areas, including stress, anxiety, depression, self-esteem, and social competence. These benefits are especially noticeable when it comes to lowering psychological stress (Fu et al., 2025).

Group cohesion represents the collective force formed among members through shared goals, emotional bonds, and collaborative needs. Physical exercise, especially team sports, serves as a vital vehicle for cultivating this cohesion. Research demonstrates a significant association between group cohesion and team success, positively enhancing collective efficacy (Carron et al., 2002; Heuzé et al., 2006). Participants in team sports face common obstacles, which are the fundamental source of unity. In school settings, peer social support significantly enhances students’ self-efficacy and physical activity engagement (Sheikh et al., 2022). Individual sports, centered on personal performance and lacking inherent collaborative elements, struggle to produce similar cohesion-boosting effects.

Psychological stress and group cohesion exhibit a bidirectional relationship. Studies on medical students reveal a negative correlation between group cohesion and stress levels (Rohe et al., 2006). A Spanish study involving 146 athletes revealed that low individualism, high social cohesion, and moderate team spirit more effectively enhance athletes’ psychological and stress management capabilities (Muñoz et al., 2023). Another survey of 20 university basketball players showed that those scoring higher on the Group Integration-Social dimension of the GEQ questionnaire exhibited lower levels of multidimensional stress related to personal, academic, and coaching pressures (Henderson et al., 1998).

Existing research confirms that physical exercise positively impacts stress reduction and group cohesion among children and adolescents, with significant variations across different sport types. However, current studies predominantly focus on the direct relationships among these factors. There are still not enough systematic studies investigating how different sports affect psychological stress in the fifth grade, and a thorough confirmation of the mediating impact of group cohesion. Accordingly, this study proposes the following hypotheses: Interactive programs are more effective than coactive programs in reducing psychological stress among children aged 10–11 years, and group cohesion may exert a mediating effect in the process through which physical exercise alleviates children’s psychological stress. This paper will test these hypotheses through an experimental design.

## 2 Experimental subjects and methods

### 2.1 Experimental subjects

A total of 144 fifth-grade children from four administrative classes at a Suzhou primary school were chosen at random to serve as experimental subjects in this study using cluster sampling. They were randomly assigned by class to either the interactive programs (*n* = 73) and the coactive programs (*n* = 71). The specific distribution is shown in Table 1. To ensure scientific rigor in the experimental design, pre-experimental comparisons were conducted to assess psychological stress and group cohesion levels between the interactive programs and the coactive programs, verifying their equivalence. Data analysis revealed no statistically significant differences (*P* > 0.05) in psychological stress or group cohesion between the two groups. Furthermore, the data exhibited normal distribution and homogeneity of variance, confirming the homogeneity of psychological stress and group cohesion levels across groups prior to the experiment.

**TABLE 1:** Distribution of experimental subjects (*N* = 144).

Group	Male	Female	Total
Interactive programs	39	34	73
Coactive programs	37	34	71
Total	76	68	144

Inclusion criteria for subjects: ➀ Fifth-grade students in stable physical and mental health with no adverse physical limitations. ➁ Students who are capable of performing experimental tasks without cognitive limitations. Exclusion criteria: ➂ Students who are frequently absent due to illness. ➃ Students with missing data from any related test before or after the experiment. The study protocol was in accordance with the Helsinki Declaration and was approved by the ethics committee of the first author’s university (Approval No. SUDA20260113H10).

### 2.2 Experimental plan

This study primarily employed an experimental research methodology. The intervention commenced in late February 2025 and concluded in late June 2025, spanning a 16-week intervention period. Experimental interventions were conducted three times weekly, each lasting 40 min. Different physical exercise interventions were implemented during physical education classes. The main learning content for the coactive programs comprised 8 weeks of track and field events and 8 weeks of gymnastics, including technical movements such as the 50-m run, standing long jump, forward roll, and backward roll, with greater emphasis on student independence. The interactive programs primary curriculum comprised 8 weeks of basketball and 8 weeks of soccer. Key basketball skills included dribbling, passing, and shooting, while soccer focused on passing, dribbling around cones, and shooting. This group emphasized collaborative learning and peer interaction during the instructional process.

### 2.3 Testing methods

Before the experiment (week 1) and after the experiment (week 16), data on psychological stress and group cohesion was collected twice. Testing was conducted in a quiet, well-lit, and distraction-free classroom at a primary school in Suzhou, fully complying with standardized testing environment requirements. Trained testing staff conducted the surveys on-site to guarantee scientific integrity in questionnaire distribution and collection. Standardized instructions clearly outlined completion guidelines, anonymity protocols, and return procedures, avoiding any suggestive guidance throughout. Testing personnel stayed on hand to answer questions without interfering with the process as students completed their answers on their own, ensuring the responses’ objectivity and authenticity.

#### 2.3.1 Psychological stress test

This study employs the “Psychological Stress Source Evaluation Questionnaire for Elementary School Students” developed by Tong Min, Shi Huijing, and other researchers. Empirical studies have demonstrated that this questionnaire possesses excellent reliability, validity, and practicality, making it a suitable tool for assessing psychological stress levels among elementary school students (Tong et al., 2023).

The scale consists of 22 items covering four dimensions: academic stress, personal physical and mental stress, family stress, as well as interpersonal stress. Participants rate psychological stress based on experienced events using a five-point scale: 0 = none; 1 = mild; 2 = moderate; 3 = severe; 4 = extremely severe. A total score ≥ 31 indicates significant psychological stress, with higher scores reflecting greater stress levels.

Before the experimental intervention, the reliability and validity of the elementary school students’ psychological stress source evaluation scale were tested. The Cronbach’s alpha coefficient of the psychological stress scale was 0.828 (> 0.7), indicating good reliability. The KMO measure of sampling adequacy was 0.754 (> 0.7), and the Bartlett’s test of sphericity was significant at < 0.001, indicating good validity of the scale.

#### 2.3.2 Group cohesion test

Questionnaire surveys and social measuring techniques are methods for evaluating group cohesion. The questionnaire survey method was used in this investigation. The Group Environment Questionnaire (GEQ), created by Carron and associates, is one of the most well-known cohesion assessment tools in sports psychology (Carron et al., 1985). In Carron’s questionnaire, the GEQ is divided into four dimensions: Individual Attractions to the Group-Task (ATG-T), Individual Attractions to the Group-Social (ATG-S), Group Integration-Task (GI-T), and Group Integration-Social (GI-S). These four aspects assess members’ perceptions of group cohesion. Subsequent studies, however, showed that elementary school pupils have difficulty distinguishing between group-oriented and individual-oriented learning. Therefore, a more appropriate scale for assessing teenage group cohesion was created by Eys et al. (2009). Given the age ranges and developmental characteristics of the participants in this study, the adolescent group cohesion scale proposed by Eys et al. was employed. This 18-item scale comprises two dimensions: Task Cohesion (8 items) and Social Cohesion (8 items), along with two negatively worded spurious items that were not included in scoring. The scale employs a nine-point rating scale, with scores ranging from 1 (“Strongly Disagree”) to 9 (“Strongly Agree”).

Before the experimental intervention, the Group Cohesion Scale was tested for reliability and validity. The Cronbach’s alpha coefficient of the Group Cohesion Scale was 0.836 (> 0.7), indicating good reliability. The KMO measure of sampling adequacy was 0.823 (> 0.7), and the Bartlett’s test of sphericity was significant at < 0.001, indicating good validity of the scale.

### 2.4 Statistical analysis

Data collected from pretest and posttest were entered into Excel sheets for organization and analyzed using SPSS 27.0 statistical software. The Youth Sport Environment Questionnaire and the Elementary Students’ Psychological Stress Sources Evaluation Questionnaire were first tested for validity and reliability. Second, changes in participants’ psychological stress ratings, group cohesiveness scores, and scale dimensions were examined using repeated measures analysis of variance (ANOVA). Lastly, Pearson correlation analysis was used to examine the associations among the study variables. Mediation analysis was then performed using Model 4 of Hayes’ PROCESS macro for SPSS. The indirect effect was estimated using 5,000 bootstrap resamples and was considered statistically significant when the 95% bootstrap confidence interval did not include zero.

## 3 Results

### 3.1 Within-group and between-group comparisons of psychological stress and group cohesion among children in different programs

To investigate the effects of different physical exercise programs on psychological stress and group cohesion among fifth-grade students, this study employed a 2 × 2 repeated measures ANOVA analysis of variance to examine the influence of group assignment and time on overall psychological stress levels and group cohesion across both groups. The analysis results are presented in Tables 2–4.

**TABLE 2:** Overall analysis of psychological stress and group cohesion across different groups (M ± SD).

Indicator	Interactive programs (*n* = 73)		Coactive programs (*n* = 71)		Time effect	Between-group effect	Interaction effect
	Pre-test	Post-test	Pre-test	Post-test			
Psychological stress	37.59 ± 12.929	27.53 ± 10.673	38.32 ± 12.308	34.32 ± 10.697	0.000**	0.044*	0.000**
Group cohesion	95.62 ± 18.444	112.01 ± 15.780	94.86 ± 18.203	101.55 ± 20.259	0.000**	0.043*	0.000**

**Indicates significant difference *p* < 0.01,

*indicates significant difference *p* < 0.05.

**TABLE 3:** Group-internal comparison of psychological stress and group cohesion among children in different groups.

Indicator	Group	Time (I)	Time (J)	Mean difference (I-J)	Standard error	*p*-value
Psychological stress	Interactive programs	Pre-test	Post-test	10.055	0.860	<0.001
	Coactive programs	Pre-test	Post-test	4.000	0.872	<0.001
Group cohesion	Interactive programs	Pre-test	Post-test	−16.397	1.826	<0.001
	Coactive programs	Pre-test	Post-test	−6.690	1.851	< 0.001

**TABLE 4:** Between-group comparisons of psychological stress and group cohesion among children in different groups.

Indicator	Time	Group (I)	Group (J)	Mean difference (I-J)	Standard error	*p*-value
Psychological Stress	Pre-test	Interactive Programs	Coactive programs	−0.735	2.105	0.727
	Post-test	Interactive Programs	Coactive programs	−6.790	1.781	< 0.001
Group cohesion	Pre-test	Interactive Programs	Coactive programs	0.757	3.055	0.805
	Post-test	Interactive Programs	Coactive programs	10.464	3.021	< 0.001

A 2 (group: Interactive Programs, Coactive Programs) × 2 (time: Pre-test, Post-test) repeated measures analysis of variance (ANOVA) was conducted on psychological stress questionnaire data. The overall psychological stress levels in both groups showed a significant main effect of time [*F*(1, 142) = 131.807, *P* < 0.001, partial η^2^ = 0.481], showing significant differences in stress levels between pre- and post-tests. Significant differences in psychological stress levels between groups were found in the main effect of group factor [*F*(1, 142) = 4.132, *P* = 0.044 < 0.05, partial η^2^ = 0.028]. Furthermore, there was a significant interaction effect between time and group [*F*(1, 142) = 24.462, *P* < 0.001, partial η^2^ = 0.147]. Simple-effects analysis showed that psychological stress levels decreased significantly from pre-test to post-test in both the interactive and coactive program groups (*P* < 0.01).

A 2 (group: Interactive Programs, Coactive Programs) × 2 (time: Pre-test, Post-test) repeated measures analysis of variance (ANOVA) was conducted on group cohesion data. Results indicated a significant main effect of time on overall group cohesion levels across both groups [*F*(1, 142) = 78.836, *P* < 0.01], confirming significant differences in group cohesion levels between pretest and post-test. A significant main effect of group was also observed [*F*(1, 142) = 4.175, *P* < 0.05], indicating significant differences in group cohesion levels between the two groups. Furthermore, a strong interaction between time and group was discovered [*F*(1, 142) = 13.937, *P* < 0.01]. Simple-effects analysis showed that group cohesion increased significantly from pre-test to post-test in both the interactive and coactive program groups (*P* < 0.01).

#### 3.1.1 Within-group and between-group comparisons of psychological stress dimensions among children in different programs

To investigate the effects of physical exercise in different programs on the dimensions of psychological stress among fifth-grade students, this study employed a 2 × 2 repeated-measures analysis of variance (ANOVA) to examine the effects of group and time factors on the levels of psychological stress dimensions in the two student groups. The results are presented in Tables 5–7.

**TABLE 5:** Repeated measures ANOVA of children’s psychological stress dimensions before and after the experiment.

Indicator	Source of variance	Type III sum of squares	Mean square	*F*-value	Significance	Partial η^2^
Academic stress	Time	45.885	45.885	8.428	0.004	0.056
	Group	22.833	22.833	0.768	0.382	0.005
	Time × Group	19.218	19.218	3.530	0.062	0.024
Family stress	Time	206.927	206.927	43.863	< 0.001	0.236
	Group	11.532	11.532	0.386	0.536	0.003
	Time × Group	13.177	13.177	2.793	0.097	0.019
Personal physical and mental stress	Time	218.675	218.675	41.639	< 0.001	0.227
	Group	106.307	106.307	4.902	0.028	0.033
	Time × Group	73.508	73.508	13.997	< 0.001	0.090
Interpersonal stress	Time	299.559	299.559	55.841	< 0.001	0.282
	Group	168.853	168.853	5.857	0.017	0.040
	Time × Group	91.018	91.018	16.967	< 0.001	0.107

**TABLE 6:** Group-internal comparisons of personal physical and mental stress and interpersonal stress among children in different groups.

Indicator	Time	Group (I)	Group (J)	Mean difference (I-J)	Standard error	*P*-value
Personal physical and mental Stress	Interactive programs	Pre-test	Post-test	2.753	0.379	<0.001
	Coactive programs	Pre-test	Post-test	0.732	0.385	0.059
Interpersonal stress	Interactive programs	Pre-test	Post-test	3.164	0.383	<0.001
	Coactive programs	Pre-test	Post-test	0.915	0.389	0.020

**TABLE 7:** Between-group comparisons of personal physical and mental stress and interpersonal stress among children in different groups.

Indicator	Time	Group (I)	Group (J)	Mean difference (I-J)	Standard error	*P*-value
Personal physical and mental stress	Pre-test	Interactive programs	Coactive programs	−0.205	0.666	0.759
	Post-test	Interactive programs	Coactive programs	−2.226	0.552	< 0.001
Interpersonal stress	Pre-test	Interactive programs	Coactive programs	−0.407	0.765	0.595
	Post-test	Interactive programs	Coactive programs	−2.656	0.604	< 0.001

Analysis of the effects of group and time factors on the levels of academic stress among the two groups of students. The analysis of intra-group effects revealed a significant main effect of the time factor on the overall levels of psychological stress in both groups [*F*(1, 142) = 8.428, *P* < 0.05, partial η^2^ = 0.056], indicating a significant difference in academic stress levels between the pre-test and post-test; the main effect of the group factor was not significant [*F*(1, 142) = 0.768, *P* > 0.05, partial η^2^ = 0.005], indicating that there were no differences in academic stress levels between the different groups. The interaction effect between time and group was not significant [*F*(1, 142) = 3.530, *P* > 0.05, partial η^2^ = 0.024].

Analysis of the effects of group and time factors on the levels of family stress among the two groups of students. Analysis of within-group effects revealed that the main effect of the time factor on the overall family stress levels of both groups was significant [*F*(1, 142) = 43.863, *P* < 0.01, partial η^2^ = 0.236], indicating significant differences in family stress levels between the pre-test and post-test; the main effect of the group factor was not significant [*F*(1, 142) = 0.386, *P* > 0.05, partial η^2^ = 0.003], indicating no differences in family stress levels between the groups. The interaction effect between time and group was not significant [*F*(1, 142) = 2.793, *P* > 0.05, partial η^2^ = 0.019].

Analysis of the effects of group and time factors on the overall personal physical and mental stress dimensions of the two student groups. There was a significant main effect of time on the levels of self-reported physical and mental stress in both groups of students [*F*(1, 142) = 41.639, *P* < 0.001, partial η^2^ = 0.227], indicating a significant difference in personal physical and mental stress levels between the pre-test and post-test. The main effect of the group factor was significant [*F*(1, 142) = 4.902, *P* < 0.05, partial η^2^ = 0.033], indicating that there were also significant differences in personal physical and mental stress levels among the different groups. Furthermore, the interaction effect between the time factor and the group factor was significant [*F*(1, 142) = 13.997, *P* < 0.001, η^2^ = 0.090]. Further simple effects analysis revealed that students in the interactive program exhibited a significant change in their levels of personal physical and mental stress between the pre- and post-experiment periods (*P* < 0.01). Students in the coactive programs also showed a decrease in their personal physical and mental stress, but this did not reach statistical significance. There was a significant difference in personal physical and mental stress levels between the two groups at the post-experiment assessment (*P* < 0.01).

Analysis of the effects of group and time factors on the interpersonal stress dimensions of the two student groups. Analysis of within-group effects revealed a significant main effect of time on the overall level of interpersonal stress for both groups of students [*F*(1, 142) = 55.841, *P* < 0.01, partial η^2^ = 0.282], indicating a significant difference in interpersonal stress levels between the pre-test and post-test. The main effect of the group factor was significant [*F*(1, 142) = 5.857, *P* < 0.05, partial η^2^ = 0.040], indicating that there were differences in interpersonal stress levels among the different groups. Furthermore, the interaction between time and group was significant [*F*(1, 142) = 16.967, *P* < 0.01, η^2^ = 0.107]. Further simple effects analysis revealed that there were significant changes in the levels of interpersonal stress among students in both groups before and after the experiment (*P* < 0.05). There was a significant difference in the levels of interpersonal stress between the two groups in the post-test (*P* < 0.01).

#### 3.1.2 Within-group and between-group comparisons of group cohesion dimensions among children in different programs

To investigate the effects of physical exercise in different programs on the dimensions of group cohesion among fifth-grade students, this study employed a 2 × 2 repeated-measures analysis of variance (ANOVA) to examine the effects of group and time factors on the levels of group cohesion dimensions in the two student groups. The results are shown in Table 8.

**TABLE 8:** Repeated measures ANOVA of children’s group cohesion dimensions before and after the experiment.

Indicator	Source of variance	Type III sum of squares	Mean square	*F*-value	Significance	Partial η^2^
Task cohesion	Time	40.475	40.475	56.788	< 0.001	0.286
	Group	7.506	7.506	3.116	0.080	0.021
	Time × Group	2.845	2.845	3.991	0.048	0.027
Social cohesion	Time	34.215	34.215	29.152	< 0.001	0.170
	Group	3.351	3.351	0.947	0.332	0.007
	Time × Group	17.070	17.070	14.544	< 0.001	0.093

Analysis of the effects of group and time factors on task cohesion in the two groups of students. The main effect of the time factor on the overall task cohesion levels of both groups was significant [*F*(1, 142) = 56.788, *P* < 0.01, partial η^2^ = 0.286], indicating a significant difference in task cohesion levels between the pre-test and post-test. There were no significant differences in task cohesiveness levels between the different groups, according to the group factor’s main effect [*F*(1, 142) = 3.116, *P* > 0.05, partial η^2^ = 0.021]. Time and group had a significant interaction effect [*F*(1, 142) = 3.991, *P* < 0.05, partial η^2^ = 0.027].

Analysis of the effects of group and time factors on the social cohesion of the two groups of students. A significant difference in social cohesion levels between the pre-test and post-test was indicated by the main effect of the time factor on the overall social cohesion levels of both groups [*F*(1, 142) = 29.152, *P* < 0.01, partial η^2^ = 0.170], while the main effect of the group factor was not significant [*F*(1, 142) = 0.947, *P* > 0.05, partial η^2^ = 0.007]. This suggests that the various groups’ levels of social cohesion were similar. Time and group had a significant interaction effect [*F*(1, 142) = 14.544, *P* < 0.01, partial η^2^ = 0.093].

### 3.2 Correlation analysis of the dimensions of psychological stress and group cohesion

The results of the correlations among the dimensions of psychological stress and group cohesion are shown in Table 9. Overall, the dimensions of psychological stress were positively correlated, and task cohesion was positively correlated with both social cohesion and total group cohesion. Task cohesion was negatively correlated with interpersonal stress (*r* = −0.183, *p* < 0.05) and total psychological stress (*r* = −0.201, *p* < 0.05). Social cohesion was negatively correlated with family stress (*r* = −0.188, *p* < 0.05). Total group cohesion was negatively correlated with family stress (*r* = −0.196, *p* < 0.05) and total psychological stress (*r* = −0.200, *p* < 0.05).

**TABLE 9:** Correlation analysis of psychological stress and group cohesion in different dimensions.

Indicator	Academic stress	Family stress	Personal physical and mental stress	Interpersonal stress	Total psychological stress	Task cohesion	Social cohesion	Total group cohesion
Academic stress	1	1	1	1	1	1	1	1
Family stress	0.528**							
Personal physical and mental stress	0.301**	0.116						
Interpersonal stress	0.351**	0.399**	0.470**					
Total psychological stress	0.776**	0.719**	0.628**	0.762**				
Task cohesion	−0.144	−0.154	−0.097	−0.183*	−0.201*			
Social cohesion	−0.073	−0.188*	−0.048	−0.096	−0.141	0.409**		
Total group cohesion	−0.125	−0.196*	−0.092	−0.162	−0.200*	0.807**	0.826**	

* Indicates significant difference *p* < 0.05,

** indicates significant difference *p* < 0.01.

### 3.3 Testing the mediating effect of group cohesion on psychological stress among children

A mediation study was carried out based on the results about the connection between stress, group cohesion, and physical activity through interactive groups. This study tests the mediational model of the effect of interactive programs on psychological stress among fifth-grade students. The hypothesized model of this study consists of three analytical steps: (1) Test coefficient C by conducting a linear regression analysis of the two variables, with interactive programs as the independent variable and psychological stress as the dependent variable; (2) Test coefficient A by conducting a linear regression analysis of the two variables, with interactive programs as the independent variable and group cohesion as the dependent variable; (3) Test coefficient B by conducting a linear regression analysis of the two variables, with interactive programs and group cohesion as independent variables and psychological stress as the dependent variable. It is hypothesized that group cohesion (M) functions as the mediating variable between psychological stress (Y) and interactive programs (X). The mediating effect of group cohesion between interactive programs and psychological stress is tested using the mediating variable testing procedure.

#### 3.3.1 Regression analysis of interactive programs and psychological stress

Interactive programs were designated as the independent variable and psychological stress as the dependent variable. A linear regression analysis was conducted on these two variables. The regression equation was statistically significant, according to the data, which revealed *F* = 14.534, *p* < 0.001, *R* = 0.305, and adjusted *R*^2^ = 0.086. The standardized regression coefficient for interactive programs on fifth-grade students’ psychological stress was −0.305, with *P* < 0.001, indicating a significant difference. This implies that psychological stress is significantly and adversely predicted by interactive programs.

#### 3.3.2 Regression analysis of interactive programs and group cohesion

A linear regression analysis was performed using interactive programs as the independent variable and group cohesion as the dependent variable. The regression equation was statistically significant, according to the results, which showed *F* = 11.995, *p* < 0.001, *R* = 0.279, and adjusted *R*^2^ = 0.071. Fifth-grade students’ group cohesion was found to be significantly correlated with interactive programs, with a standardized regression coefficient of 0.279 (*p* < 0.001). This suggests that interactive programs significantly and positively predict group cohesion.

#### 3.3.3 Regression analysis of group cohesion and psychological stress

Group cohesion was treated as the independent variable and psychological stress as the dependent variable. These two variables were subjected to a linear regression analysis. The linear regression equation was statistically significant, according to the results, which showed *F* = 5.895, *p* < 0.05, *R* = 0.200, and adjusted *R*^2^ = 0.033. The standardized regression coefficient for group cohesion on psychological stress among fifth-grade students was −0.200, with *p* = 0.016 < 0.05, indicating a significant difference. This suggests that group cohesion significantly and negatively predicts psychological stress.

#### 3.3.4 The mediating role of group cohesion in psychological stress in interactive programs

First, virtual variables were created based on the experimental groups, with physical exercises in interactive programs coded as 1 and those in coactive programs coded as 0. Using the Process macro in SPSS 27.0, a mediation analysis was conducted based on the Bootstrap method. Model 4 was selected, and a 95% confidence interval was set. In this mediation analysis, the dummy variable served as the independent variable, psychological stress as the dependent variable, and group cohesion as the mediating variable. The total psychological stress score, the total group cohesion score, and the scores of each dimension were calculated by taking the difference between the post-test and pre-test scores, which were standardized prior to analysis, in order to reduce the interference of individual differences and guarantee more accurate results.

**FIGURE 1:**
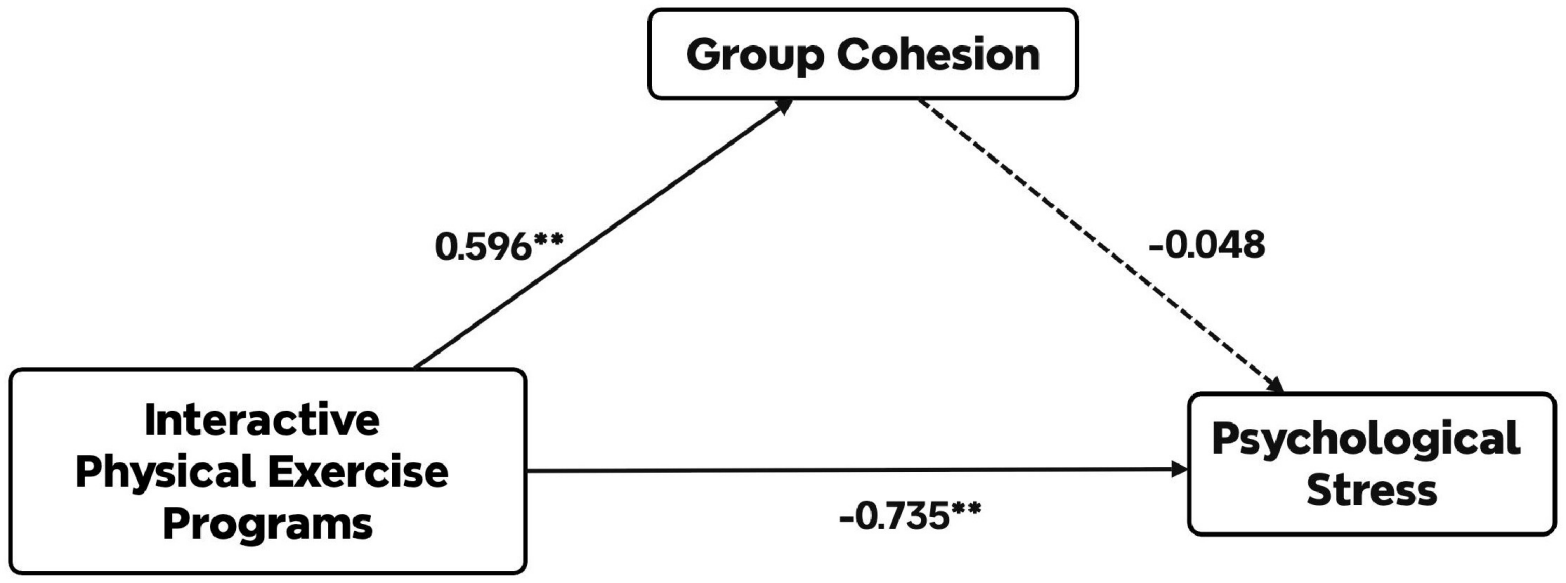
The path diagram of the regression coefficients for the mediating effect of group cohesion. ** Indicates *p* < 0.01, representing a highly significant difference.

According to the analysis, interactive programs have a significant overall impact on psychological stress (β = −0.764, SE = 0.154, 95% CI [−1.069, −0.459]). In the mediation model, after controlling for the mediating variable of group cohesion, the direct effect of interactive programs on psychological stress remained significant (β = −0.735, SE = 0.162, 95% CI [−1.056, −0.414]), accounting for as much as 96.2% of the effect. In contrast, the mediating effect through group cohesion was not significant (β = −0.029, SE = 0.052, 95% CI [−0.145, 0.067]); the confidence interval for this effect included 0, and its proportion of the total effect was only 3.8%. This indicates that the mediating influence of group cohesion on the relationship between interactive programs and psychological stress is not significant. In this study, the impact of interactive programs on psychological stress is primarily mediated through a direct pathway (as shown in Figure 1).

## 4 Discussion

### 4.1 Analysis of the current status of psychological stress and group cohesion among children

According to pre-test results, students in the interactive programs had a total psychological stress level of 37.59, and those in the coactive programs had a score of 38.32. The scores of both groups were significantly higher than the threshold of 31 points established by the Questionnaire for Assessing Sources of Psychological Stress in Elementary School Students, suggesting that students in this age group generally experience a certain level of psychological stress. Mental health problems like stress and anxiety are already common among elementary school students, according to a large-scale quantitative study on psychological stress among primary and secondary school students by Sood et al. (2024). Psychological stress has become a prevalent issue among children in this age group. These pressures not only impact their academic performance and overall wellbeing, but they may also have a detrimental impact on their long-term physical and mental health development. In a study conducted by Zhu et al. that involved 1,533 adolescents in eastern China, it was also observed that academic pressure and anxiety are significantly positively correlated among 10–12-year-olds (Zhu et al., 2021). This correlation is stronger than that observed in other age groups. The patterns of juvenile psychological development and changes in the social environment are closely associated with the prevalence of psychological stress among students at this stage. On the one hand, students are required to contend with more intricate learning and higher academic standards as academic difficulty increases and expectations regarding future education begin to arise, resulting in a continuous accumulation of academic stress. On the other hand, children between the ages of 10 and 11 undergo a rapid development of self-awareness and begin to prioritize peer evaluation, their own performance, and family expectations. This leads to a substantial increase in both internal psychological stress and interpersonal stress. A study conducted among school-aged children in a specific region of Nigeria has shown that peer relationships are substantially correlated with self-awareness and social adaptation. The children’s levels of self-awareness and social adaptation are higher when the peer relationships are strong (Nwauzoije, 2023).

Pre-test data from this study indicate that the interactive programs had a total group cohesion score of 95.62, while the coactive programs had a score of 94.86. Both groups’ scores were within the moderate range, which suggests that pupils in this age group already have a sense of group belonging, given the nine-point scale’s scoring range. Both kinds of programs in this study improved children’s group cohesion in terms of intervention efficacy, with the interactive programs seeing a noticeably higher increase than the coactive programs. This outcome demonstrates the beneficial effects of physical exercise in interactive programs treatments on children’s group cohesion. Zheng Jing discovered that the basketball group showed more improvements in group cohesion than the aerobics group in an experimental research of elementary school students (Zheng, 2022). Children’s sense of belonging to the group and the attraction among members can be substantially improved by establishing shared goals and reinforcing scenarios of peer collaboration. Compared to coactive programs, interactive programs are more effective in fostering an understanding of the relationship between the individual and the team among children by facilitating frequent peer communication, negotiation, and mutual assistance. Consequently, the level of group cohesion is increased. Bruner’s research also suggests that adolescents’ personal development in physical activities is significantly influenced by sports teams with strong cohesion (Bruner et al., 2014).

### 4.2 The effect of different physical exercise programs on psychological stress among children

#### 4.2.1 The effect of different physical exercise programs on the overall psychological stress among children

The findings indicate that, following a systematic experimental intervention, physical exercise can effectively reduce stress levels in 10–11-year-old schoolchildren, while interactive programs are even more effective in adjusting children’s emotions and alleviating psychological stress. Similar findings have been reported in previous studies. Gould and Carson note that the development of social skills through sport is a key pathway for adolescents to cope with stress and build positive psychological resources (Gould and Carson, 2008). Research by Cao et al. indicates that a positive attitude toward exercise and moderate physical activity are significantly associated with lower levels of perceived stress (Cao et al., 2021). Haugland et al. found that physical activity acts as a significant buffer between school-related stress and physical and psychological distress in adolescents (Haugland et al., 2003). Team sports offer profound benefits that extend beyond physical health, having a positive impact throughout the entire lifespan (Larsen et al., 2025).

Physical exercise in interactive programs was significantly more effective at reducing psychological stress levels among primary school students than in the coactive programs group. This may be because interactive programs place greater emphasis on social interaction and teamwork, requiring students to communicate during exercise and cooperate to achieve shared goals. Erikson’s psychosocial development theory proposes that the core of development in children aged 6–12 lies in the formation of a sense of diligence and self-efficacy through learning and group activities (Erikson, 2021). At this stage, children begin to place great importance on the evaluations of their peers and teachers and develop a sense of self-worth through collaborative activities. Through cooperation, division of labor, and immediate feedback, interactive activities give kids the chance to get positive social feedback and peer recognition, strengthening their sense of self and emotional stability. In contrast, coactive activities, due to their lack of sustained interpersonal collaboration and social feedback, play a relatively limited role in fostering positive psychological experiences. Furthermore, Selman’s role-taking theory suggests that children aged 10–11 are in the stage of self-reflective role-taking and reciprocal role-taking (Selman and Byrne, 1974). At this age, children start noticing their own behaviors from other people’s perspectives and constantly modify their emotional reactions and behavioral strategies in social situations. Interactive programs’ cooperative communication, shared responsibilities, and peer interaction provide kids real-world opportunities to experience social roles and adopt new perspectives, which improves their social flexibility and emotional control. In contrast, coactive programs involve a lower degree of interpersonal interaction and are therefore less able to fully satisfy children’s psychological needs for social development. Pueyo et al.’s research shows a strong negative correlation between psychological issues and physical activity (Pueyo Gutiérrez-Rivas et al., 2025). Teenagers who play team sports show significantly lower stress levels than those who play individual sports. Similarly, team sports are linked to lower physical anxiety symptoms than individual sports, according to research by Dimech and Seiler (2010).

#### 4.2.2 The effect of different physical exercise programs on the dimensions of psychological stress among children

The results show that different types of physical activity have varying effects on the different aspects of psychological stress in children aged 10–11. There were no apparent distinctions between the different kinds of exercise in terms of academic and family stress, despite the fact that stress levels decreased in both student groups after the experiment. This may be due to the fact that factors including academic workload, parenting approaches, and the external environment have a bigger impact on academic and family stress. As a result, it is doubtful that improvements in exercise solely will result in meaningful intervention benefits.

A simple effects analysis showed that the interactive programs group outperformed the coactive programs group in terms of both personal physical and mental stress and interpersonal stress. This implies that physical activity in interactive programs has more benefits to fostering kids’ psychological adjustment. Cooperation, communication, and coordination are often emphasized in interactive programs. Children’s mental health and social experiences are improved more successfully when they engage in physical activity because it gives them more opportunities to express their feelings, receive more peer support, and engage in positive social interactions. By contrast, while coactive programs can also encourage participation in physical activity, their impact on students’ psychosocial development is limited due to their relatively low level of interactivity. In their systematic review, Eime et al. noted that sports involving strong teamwork and interaction are more likely to result in positive psychosocial benefits than physical activity alone (Eime et al., 2013). According to a recent review by Wade et al., team sports have greater and more reliable psychosocial advantages than individual sports, with social support and a sense of peer belonging acting as important mechanisms for enhancing mental health (Wade et al., 2026). Similarly, a long-term study by Moeijes et al. discovered a strong positive relationship between kids’ psychosocial wellbeing and their involvement in sports. In particular, interactive sports are better at preventing internalizing problems in kids and encouraging the growth of prosocial behavior (Moeijes et al., 2018).

Consequently, this study suggests that the benefits of interactive physical exercise programs extend beyond general stress relief. More importantly, they can have a positive impact on the social confidence and internal psychological state of pre-adolescent children. While personal physical and mental pressures, as well as interpersonal pressures, are more easily impacted by the quality of social interaction, peer relationships, and emotional experiences during physical activity, academic and family pressures are heavily influenced by external environmental factors. Interactive programs provide children with positive social support and psychological experiences during exercise by increasing opportunities for cooperation, communication, and emotional connection. This helps to better support children’s mental health development. This further demonstrates how the psychological advantages of exercise are closely related to the social interaction aspects of the activity as well as the physical activity itself.

### 4.3 The effect of different physical exercise programs on group cohesion among children

The findings indicate that, following a 16-week intervention, both interactive and coactive exercise programs were able to enhance group cohesion among 10–11-year-old children compared to pre-intervention levels, with the interactive programs showing significantly greater improvements than the coactive programs.

This conclusion is also supported by earlier studies. Kwon’s meta-analysis showed that team-building interventions in interactive team sports can effectively enhance group cohesion, with the greatest improvement observed in task-oriented cohesion (Kwon, 2024). Gu et al., researching athletes in highly interactive team sports such as football and basketball, found that interactive scenarios play a role in activating cohesion (Gu et al., 2022). Through long-term empirical study on interactive team sports like football and hockey, McEwan et al. discovered that cooperative behavior greatly increases task cohesiveness and, through task cohesion’s mediating effect, raises team performance satisfaction (McEwan, 2020).

This finding may be linked to differences between various types of physical exercise in terms of task collaboration and emotional interaction. As a comprehensive construct that integrates task orientation and emotional bonding, group cohesion depends not only on the achievement of shared goals, but also on interpersonal interaction and emotional support from group members. Interactive activities center on teamwork. In order to achieve a common goal, students must divide tasks, communicate in real time, and support each other throughout the exercise. This collaborative experience not only strengthens task cohesion but also fosters emotional bonds between members, thereby enhancing overall group cohesion. Previous research has indicated that team sports can provide structured social support, enhance a sense of belonging and reduce feelings of loneliness, and that such social connections serve as an important psychological resource for stress relief (Eime et al., 2013). According to Raut’s research, the social support that comes from peer cooperation and shared experiences is a major factor in the beneficial impacts of team sports on mental health (Raut, 2024). Furthermore, in a 10-month study, Elbe et al. found that, compared to individual sports, team sports were more effective in maintaining children’s enjoyment of physical activity and social cohesion among 8–10-year-olds (Elbe et al., 2017).

By contrast, coactive projects place greater emphasis on the completion of individual tasks and the achievement of personal goals, with relatively limited interpersonal interaction and emotional exchange among students. The lack of continuous collaboration and immediate social feedback means that such projects have a relatively weaker effect in fostering task cohesion and social cohesion, even though students may develop a certain sense of group identity through their joint participation. Their sense of group identity requires both an awareness of purpose derived from shared goals and an understanding of belonging fostered by peer interaction. As a result, interactive physical activities are more appropriate for satisfying students’ psychological development needs at this point, leading to more significant improvements in group cohesion.

### 4.4 Analysis of the non-significant mediation effect of group cohesion

The results of this study show that group cohesion does not significantly mediate the relationship between interactive physical exercise and psychological stress in children aged 10–11, which is contrary to the original research hypothesis. The mediating hypothesis of group cohesion was chosen for this study mainly because it was supported by relevant empirical evidence and current theory. According to earlier research, physical activities with a high level of involvement and collaboration can successfully foster the development of group cohesion through shared objectives, peer connection, emotional exchange, and cooperative experiences. According to Carron et al., task cohesion and social cohesion are the two main components of group cohesion, which is a dynamic process based on shared objectives and emotional ties (Carron et al., 1985). According to research on teenage groups by Bruner et al. and Eys et al., cooperative experiences, interpersonal contacts, and the distribution of duties in team sports are important elements in promoting the growth of adolescent group cohesion (Bruner et al., 2014). At the same time, existing research has also found a significant negative correlation between group cohesion and psychological stress. By strengthening social support, a sense of belonging, and a sense of collective identity, higher levels of group cohesion can partially reduce an individual’s stress reaction and improve their emotional state. Rohe et al. found that strong group cohesion can effectively reduce individuals’ perceived stress and negative emotions (Rohe et al., 2006). Higher levels of group cohesion can also improve collective efficacy and team stability, according to a study on professional basketball players by Heuzé et al. (2006). Additionally, the interactive programs used in this study, like football and basketball, naturally emphasize cooperation, communication, teamwork, and the accomplishment of common objectives. They give pupils more chances for social engagement and emotional sharing than coactive programs. Therefore, this study hypothesizes that group cohesion may be a crucial psychological mechanism via which interactive physical exercise training affects psychological stress based on theoretical reasoning and practical situations.

However, the findings of this study indicate that, although the interactive programs significantly enhanced the students’ group cohesion and effectively reduced their psychological stress—particularly demonstrating a more pronounced improvement in the dimension of social cohesion. This did not translate into an effective mediating pathway for alleviating psychological stress. This may be related to a mismatch between the characteristics of children’s psychological stress and the mechanisms through which group cohesion operates. The main characteristics of psychological stress in children aged 10–11 are its immediacy, situational nature, and short-term duration (Compas, 1987). These sources of stress typically come from everyday situations such as academic workload, classroom performance, peer conflicts and teacher evaluations, and often require rapid, immediate emotional regulation and stress relief. By contrast, group cohesion is more often manifested as a long-term, stable source of social support. The social support buffer model proposed by Cohen and Wills proposes that the buffering effect of social support against stress usually depends on long-term, stable interpersonal bonds and sustained emotional support, rather than producing immediate regulatory effects in the short term (Cohen and Wills, 1985). As a result, even though the children’s social and task cohesion levels were significantly increased through the interactive program, this improvement in cohesion was insufficient to have a stabilizing moderating effect on psychological stress throughout the brief intervention period. Furthermore, from the perspective of developmental psychology, children’s social cognitive abilities and coping methods for stress are not yet fully mature, and their capacity to proactively understand and utilize social support resources is still relatively limited. Consequently, although the interactive programs significantly enhanced the students’ social cohesion, fostering closer emotional bonds and a greater sense of team belonging during the activities, children may not necessarily be able to proactively and effectively transform this interpersonal group support into cognitive resources for alleviating psychological stress. Furthermore, the findings of this study also indicate that physical exercise within interactive programs has a significant direct impact on psychological stress. Regular physical exercise can produce antidepressant and anxiolytic effects and reduce stress sensitivity through stress adaptation and neurochemical regulation (Pluhar et al., 2019). For children aged 10–11, this immediate physiological and emotional regulation may have a more direct impact on psychological stress than long-term social support mechanisms based on group cohesion, thereby weakening the mediating effect of group cohesion to some extent.

The role of interactive physical exercise in reducing psychological stress may also be impacted by a number of different psychological and physiological mechanisms, in addition to group cohesion. Psychological resilience may be one potential latent variable. Through the psychological experience of overcoming repeated problems, physical exercise can gradually enhance students’ psychological resilience, which includes their adaptability, frustration tolerance, and emotional recovery capability when faced with stress. A meta-analysis by Lin et al. found that psychological resilience plays a significant mediating role between physical activity and mental health (Lin et al., 2024). According to Xu and Tian’s research, group cohesion can help athletes develop psychological resilience through routes including self-identity and social support (Xu and Tian, 2025). Consequently, the teamwork, emotional support and sense of belonging fostered by interactive projects may not directly alleviate psychological stress, but rather indirectly influence students’ mental wellbeing by enhancing their psychological resilience. Additionally, self-efficacy might be a crucial psychological mechanism by which exercise affects psychological stress. Self-efficacy is a person’s subjective evaluation of their capacity to carry out a specific task. Exercise-related skill development, goal accomplishment, and positive reinforcement can increase students’ self-confidence and strengthen their psychological resilience in handling stressful situations. According to earlier studies, physical activity is a crucial strategy for raising students’ self-efficacy in sports and can successfully increase both group cohesion and self-efficacy (García Guillén et al., 2025). Self-efficacy acts as a full mediator in the relationship between physical exercise and emotional regulation (Mu et al., 2024). High self-efficacy students are more likely to actively participate in cooperation and group interactions, which further strengthens their sense of collective support and reduces psychological stress. They are also more likely to understand difficult circumstances positively.

As a result, this study suggests that the effects of interactive physical exercise on psychological stress may be the consequence of the interaction of several psychological systems rather than a single mediating mechanism. In order to create a more accurate multiple-mediation model and better understand the underlying mechanisms through which interactive physical exercise affects children’s psychological stress, future research could include variables like psychological resilience, self-efficacy, and emotional regulation.

## 5 Conclusion

Physical exercise can effectively alleviate psychological stress and improve group cohesion in children aged 10–11 years. Notably, interactive programs are more effective than coactive programs in promoting both outcomes. The impact of interactive programs on psychological stress is primarily achieved through a direct pathway, with group cohesion not playing a significant mediating role.

## 6 Limitations

The participants in this study were limited to fifth-grade students from a small number of elementary schools in a single region. The sample’s geographic distribution and socioeconomic background were relatively concentrated, and the study lacked coverage of students from both urban and rural areas as well as schools of varying educational quality. Therefore, the generalizability of the findings is limited. Additionally, the sample size did not fully account for the high requirements of mediational analysis, which may have diminished the statistical power of the tests and made it difficult to detect the potentially weak association of the mediating effect of group cohesion. It is expected that future research will encompass a broader sampling area and a larger sample size, extending the intervention period to 6 months or even a year or more to evaluate the long-term dynamic relationship between group cohesion and variables such as psychological stress. The primary method of variable measurement in this study was student self-reports, which were not cross-validated with sources such as teacher evaluations and parental feedback. This may have resulted in data distortion due to self-reporting biases among fifth-grade students. In order to improve the reliability and validity of the data, future research could integrate qualitative methods, such as interviews and observations, to combine quantitative data with qualitative findings. This study focused only on the mediating role of group cohesion and did not explore its interactions with other potential mediating variables or chain mediation effects. Future research could endeavor to incorporate additional variables in order to investigate the mechanisms by which physical exercise influences psychological stress through alternative pathways.

## Data Availability

The raw data supporting the conclusions of this article will be made available by the authors, without undue reservation.
